# Improving school-age nutrition and school performance through amaranth plus flaxseed food product distribution in Sidama, Ethiopia: a study protocol

**DOI:** 10.1080/16549716.2025.2556087

**Published:** 2025-10-15

**Authors:** Alemselam Zebdewos Orsango, Aberash Eifa Dadhi, Mekdes Tigistu, Tizita Gebeyehu Yismaw, Mehretu Belayneh Dinage, Ingunn Marie S. Engebretsen

**Affiliations:** a School of Public Health, College of Medicine and Health Sciences, Hawassa University, Hawassa, Ethiopia; b Centre for International Health, Department of Global Health and Primary Care, University of Bergen, Bergen, Norway; c Department of Public Health, Institute of Health Science, Wollega University, Wollega, Ethiopia; d School of Economics and Finance, Faculty of Commerce, Law and Management, University of Witwatersrand, Johannesburg, South Africa

**Keywords:** Amaranth, flaxseed, nutritional anaemia, high-density lipoprotein, iron

## Abstract

Primary school-age children are particularly vulnerable to undernutrition, especially anaemia and underweight. School feeding programs in food-insecure areas aim to reduce undernutrition, but many vulnerable children in Ethiopia have not benefitted due to inadequate food quality and sustainability challenges. Exploring underutilized nutrient-rich foods may help address the shortage of energy-dense supplements for these programs. Amaranth, with its leaves consumed as a vegetable and seeds used like cereals, has shown superior nutrient content when compared to maize. Previous interventions with amaranth in younger children demonstrated a significant reduction in anaemia prevalence. This study aims to assess nutritional health and to reduce undernutrition among school children by promoting amaranth-plus-flaxseed food from locally grown, standardised foods in Sidama, Ethiopia. Under this research project, the following three study designs will be undertaken: a laboratory-based food analysis study, a cross-sectional study, and an experimental pilot study. In the pilot study, we aim to observe a weight increase of 0.5 kg, a haemoglobin increase of 0.5 mg/dl, and a reduction in school dropout. This protocol outlines the detailed procedure of the school intervention from food formulation to the pilot study. The outcomes generated from this project will provide policymakers with valuable insights to consider alternative approaches for school intervention.

## PAPER CONTEXT

- **Main findings:** The primary finding of this proposal will be the impact of formulated food on the nutritional status and academic performance of school-age children. This research aims to establish a clear link between the consumption of nutrient-rich formulated foods and improvements in both health and educational outcomes.
- **New knowledge:** This study will contribute new knowledge regarding: The specific nutrient content of the formulated food being tested, the relationship between the intake of formulated food and the nutritional status of school-children in the targeted area and insights into how improved nutrition through formulated foods may enhance academic performance among school-aged children.
- **Global Health impact for policy and action:** If the intervention proves successful, it could lead to the development of cost-effective, nutrient-rich school snacks designed to combat malnutrition and improve educational performance among children. This could inform policy decisions at local, national, and global levels, promoting initiatives that prioritize nutritional interventions in schools. Ultimately, this could help address significant public health challenges related to child malnutrition and educational disparities, paving the way for healthier, more academically successful future generations.

Preprint: https://medrxiv.org/cgi/content/short/2025.02.05.25321679v1

## Background

Under-nutrition is a major cause of childhood morbidity and mortality in low-income countries (LICs) [1]. It is an intergenerational problem because preconception and intrauterine factors contribute to child growth and development [2–4]. Under-nutrition contributes to compromised cognitive development [5–7] and affects learning, productivity, and creativity [8–10]. In Ethiopia, the poor, who constitute the majority, are unable to access adequate amounts of nutrient-rich foods to meet daily dietary requirements [10–14].

A synthesis of review studies from low- and middle-income countries (LMICs) indicates that information about the nutritional status of school-aged children is not well stated [14,15]. In the northern part of Ethiopia, a study indicated that 79.5% of school-aged children had at least one micronutrient deficiency in 2014 [15–17]. Anaemia is one of the biggest nutritional problems in the school-age going group, affecting 23% of the group [17,18]. In 2022, a multi-centre cross-sectional study showed that the prevalence of wasting accounted for 18% of primary school-aged children in Ethiopia [18,19]. A study from the capital city of Ethiopia indicated that 58% of school-aged children had low levels of high-density lipoprotein (HDL) [19,20].

Causes of nutritional anaemia in children are multifactorial and associated with various micronutrient deficiencies. Deficiencies of iron, vitamin B12, and folic acid are the most common causes of anaemia [20,21]. Despite its importance, the consumption of animal source foods and leafy vegetables in children is low in Ethiopia [11,12,21,22]. Further, the consumption of animal-source food and leafy vegetables related to the magnitude of folic acid and Vitamin B12 deficiencies in schoolchildren has not been explored well in Ethiopia.

The global evidence suggests that school feeding interventions have a positive impact on both nutritional status and school attendance among children. Systematic reviews of various studies indicate that school feeding interventions can lead to improved growth outcomes and increased school attendance and participation [22]. School feeding in Ethiopia began in 1994, targeting food-insecure areas by providing one hot meal composed of corn-soya blend, vegetable oil, and salt. In 2012, Home-Grown School Feeding (HGSF) programs were introduced by the World Food Program (WFP) to cover the majority of school children [23]. The HGSF is a school feeding model that provides children in schools with safe, diverse, and nutritious food sourced locally from smallholders [24].

The Ethiopian government listed School Health Nutrition (SHN) in the new Education Sector Development Plan V in 2016 and launched the SHN strategy based on the Focusing Resources on Effective School Health (FRESH) approach [25–27]. That approach aims to improve food access and educational achievement of schoolchildren through health and nutrition interventions and programs across schools in Ethiopia [27,28]. But the World Food Program (WFP) report from 2013 to 2018 indicated the coverage of school feeding programs to be only 9% of the school children in Ethiopia [24]. In 2022, FAO evaluated the home-grown school feeding program by stakeholders. The evaluation highlighted the importance of building the capacities of smallholder farmers to produce locally available, sufficient, good-quality, and nutritious food to sustain the HGSF programs [29]. Moreover, the five-year school feeding programme, which is in the Afar and Oromia regions in Ethiopia (2019–2024), is funded by the McGovern-Dole International Food for Education and Child Nutrition Program of the United States Department of Agriculture (USDA). This program was evaluated from 2020 to 2022, and the evaluation report indicated that the program is beneficial for food-insecure households. However, sustainability is a problem as the food is imported from outside Ethiopia [30].

Regarding the effect of school feeding programs, a study from southern Ethiopia found that school feeding programs decrease school absenteeism and dropout [31,32]. The effect of school feeding on nutritional status is controversial in Ethiopia. Studies on school feeding programs found that the school feeding programs with a diverse diet have a significant effect on the nutritional status of school children [33], but a study from the rural part of Sidama region showed that the school feeding program had no effect on the anthropometric and haemoglobin, measures of school children [34]. The possible reason observed under this study was that the quality of school meals provided to children was possibly not optimal to advance the nutritional status of children, since the school feeding program provided a daily hot meal prepared from 150 g of dry cereals and beans in different forms [34]. Also, studies of costs and cost-efficiency of school-based feeding programs in food-insecure areas showed that fortified biscuits provided the most cost-efficient option in terms of micronutrient delivery. This study recommends that costs, effects, and sustainability should be considered carefully when designing school feeding interventions [32].

Amaranth is one of the few plants where the leaves are eaten as a vegetable, while the seeds are used in the same way as cereals [35]. The seeds contain high levels of protein that are unusually complete for plant sources and are a good source of dietary fibre and minerals such as iron, zinc, copper, and manganese [36–38]. Amaranth grain has the characteristic of producing high yields even in relatively dry areas within a short period. Grain amaranth can be used as seeds or flour to make products such as cookies, cakes, pancakes, bread, muffins, crackers, pasta, and other bakery products. Although the grain has quality nutrients, it also has a high concentration of phytic acid that can reduce the bioavailability of nutrients [39]. To solve this problem, our previous study identified that homemade processing, such as soaking and germination of amaranth grain could reduce the phytate level, thus increasing the bioavailability of nutrients. This finding has been supported by research from Kenya [37,40].

In our previous work, we found that amaranth grain, which grows as a wild plant, has a higher iron content compared to the commonly consumed staple food maize [37]. Further, to understand the absorption of iron from the gut, we did a six-month intervention study on children who had anaemia. The results indicated that the prevalence of anaemia decreased in the amaranth group. But, we didn’t identify a significant weight or height change in the amaranth group [36]. We believe this was due to the amount of energy content in the formulated bread.

Further, we found that the consumption of fish or seafood in the study area is almost null [12].

In Ethiopia, omega-3 fatty acid food sources are scarce and expensive, so the consumption of these food sources is low. Flaxseed oil could be one of the alternatives to improve the intake of energy as well as the omega-3 fatty acids. Flaxseed is one of the oil plants that grows widely, and its contribution to supplementing omega-3 fatty acids is not well recognized in Ethiopia.

Integrating amaranth and flaxseed is expected to enhance the micronutrient content, particularly iron, essential fatty acids, and amino acids, thereby improving the nutritional quality of school feeding snacks. Additionally, since these crops are locally grown, they offer a cost-effective solution. Investigating underutilized and nutrient-dense food sources may help address the lack of energy-rich and nutrient-dense food options for school feeding initiatives. The primary goal is to formulate a nutrient and energy-dense snack, assess the nutritional quality of the formulated snack, the nutritional health of school children, and examine the impact of providing a food product made from amaranth and flaxseed on reducing undernutrition among children in Sidama, Ethiopia.

## Methods

### Study settings and designs

The study will be conducted at Shebedino Woreda rural public primary schools in Sidama Regional State, Ethiopia. The Sidama region has 36 districts (6 urban districts and 30 rural districts), including the Hawassa city administration. The total population of the Region is 4.4 million. Shebedino is one of the districts (woreda) and has a rural and urban wing; the rural wing of the woreda has 26 kebeles. There are 35 governmental schools in the rural wing of the woreda: from these 33 are primary and 2 are secondary schools. The woreda’s rural wing is divided into 26 kebeles. 33% of the woreda is classified as dega (highlands), and the remaining 67% is woyinadega (midlands), and rainfall is usually abundant at 900–1100 mm per year as the long-term mean. Inset (Kocho) and maize are the people’s main foods. Coffee, inset, fruits, avocado, mango, papaya, banana, vegetables, and chat are the most prevalent income crops in this area. Shebedino woreda has one hospital, six health centres, twenty-three health posts, and a total of 401 health professionals, including 76 health extension workers.

**Figure 1.**
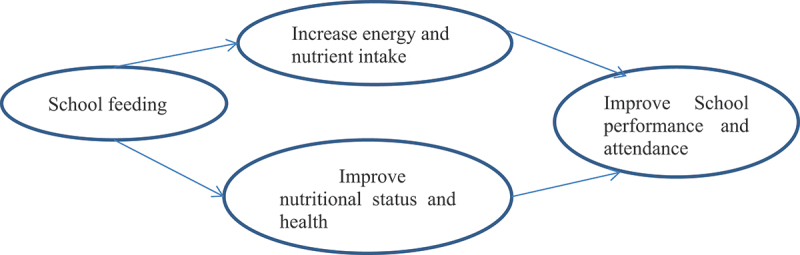
Conceptual framework.

Three types of designs will be utilized, consisting of four work packages (WPs), each featuring a distinct study design that is interconnected. A laboratory-based experimental study will develop the experimental bread to assess its nutrient content and safety levels; this study will contribute to work package III. A cross-sectional study design will examine the prevalence of undernutrition among school children in the area, helping to highlight the significance of school feeding programs by identifying rates of undernutrition and food insecurity (Table 1). Work packages III and IV will involve an experimental pilot study to evaluate the formulated nutrient-rich and energy-dense school snack on the nutritional and school performance of children attending primary school (Figure 1).

**Table 1.** Study design and outcome.

Study	Design	Outcome
Work package one	Laboratory-based	Acceptability, Nutrient content
Work package two	Cross-sectional study	Anaemia, nutritional status and school health and performance
Work packages three and four	Pilot trial	Nutritional anaemia, HDL, growth and educational outcome

The objectives of each of the three work packages (WPs) are outlined below, followed by a description of each.

- To formulate an amaranth plus flaxseed-based energy-dense and micronutrient-rich food product for school children 7–10 years of age in rural schools of Sidama, Ethiopia and conduct acceptability testing (WP 1).
- To assess the prevalence of and factors associated with under-nutrition in school children 7–10 years of age in rural schools of Sidama, Ethiopia (WP 2), and
- To assess the effect of an amaranth-plus, flaxseed-based, energy-dense and micronutrient-rich food product distribution on nutritional status in school children 7–10 years of age in rural schools of Sidama, Ethiopia (WP 3).
- To investigate the impacts of amaranth plus flaxseed food products on the education outcomes of school-age children 7–10 years of age and its cost-effectiveness in Sidama, Ethiopia (WP4).

## Work package 1

In this WP, amaranth, flaxseed, chickpea, and emir wheat will be used for the formulation of an energy and nutrient-dense recipe for the experimental school feeding pilot. Home-level processing will be applied to the amaranth grain to reduce the phytate level. Amaranth grain will be soaked in water by adding 5 ml of lemon juice per 100 ml of water for 24 hours and germinated for 72 hours. After sun-drying, it will be roasted and milled with a local electrical mill [35]. The chickpeas will also be soaked for 24 hours, dried, roasted, and milled. The flaxseed, emmer wheat, oats, and maize will be sorted, washed, dried, roasted, and milled.

### Flour blend formulation

The flour blends from prepared flours will be formulated for the preparation of a recipe for standardised food preparation. The grain ratio will assess the combination that has the highest nutritive value (nutrients and energy), the lowest anti-nutrient ratio to nutrient and acceptable taste, texture and preparation qualities.

### Acceptability

A consumer acceptability test will be done at the laboratory and community levels by using nine-point hedonic tests. Community acceptability tests will be done with school children.

### Nutrient analysis

The nutrient composition, including minerals (iron, zinc and copper), heavy metals, amino acids, fatty acids, and dry matter, will be assessed by the Institute of Marian Research (IMR) accredited by the Norwegian Accreditation with registration number TEST 050. Carbohydrate content will be determined by estimating the difference by calculating crude protein, crude fiber, total ash andcrude fat (Carbohydrate% = 100 - (fat %+protein % + ash % + moisture %). The total energy content (calories) of the food will be calculated using its fat, carbohydrate, and protein content. We’ll use standard conversion factors (Atwater factors) to do this: 4 calories per gram for protein and carbohydrates, and 9 calories per gram for fat. The formula we will use is: calories per 100 grams = (4 *carbohydrates +4 *proteins +9 * fats). To ensure the food is safe, we’ll test for harmful microorganisms like *E. coli*, Coliform bacteria, and total bacteria count using standard laboratory methods at the Hawassa University food and nutrition laboratory centre. Once we confirm that the food meets safety and palatability standards according to FAO and WHO guidelines for contaminants and toxins, the intervention will be determined [41,42].

## Work package 2

The cross-sectional survey will investigate the nutritional status among 7–10-year-old primary school-going children and estimate the prevalence of undernutrition and associated factors. The study will include grade 1–2 actively learning school children (7–10 years old) with parents or guardians at selected primary schools. Those who are seriously ill or have chronic illness, have severe malnutrition, have a history of any known allergy, are participating in other trials, are unable to take solid food, and grade 1 or 2 children aged > 10 years will be excluded from the cross-sectional survey.

### Sample size calculation WP 2, 3 and 4

Sample size was calculated in two steps for the cross-sectional survey part (WP 2) and the experimental part (WP 3).

Sample size for the cross-sectional survey was computed using Epi Info 7.2.5.0 statistical software by considering a single population proportion with the assumptions of an alpha value of 5%, a confidence interval (CI) of 95%, a margin of error of 5%, for each outcome variable and the largest sample size was taken. Sample size ***n*** = [DEFF*Np (1-p)]/[(d^**2**^**/Z**^**2**^_1-α/2_*(*N*-1) + p*(1-p)]. We calculated the sample size for each outcome variable and ultimately chose the largest sample size needed, which was 374. This was determined based on the factor associated with undernutrition, specifically the prevalence of low high-density lipoprotein (HDL), estimated at 58.4%.

The previously reported value of the intra-cluster correlation coefficient (ICC) factor from the study reported on summarized minimum intra-cluster correlation coefficient (ICC) estimates for pupil health outcomes from school-based cluster randomized trials (CRTs) across the world region was 0.031 (0.01 to 0.08) we took the minimum value0.01 [43]. To account for the design effect, the sample size was multiplied by 1.59. The design effect was calculated with the assumption of an equal size of clusters. Using an intra-cluster correlation coefficient value of 0.01, (DIEF = 1+ [(*n*-1) ICC]), where ‘n’ is the average class size, which is 60 students per class, accordingly, the adjusted sample size was 595. Accounting for a 10% non-response rate, the final sample size estimated was 654 participants from 36 classes in 6 schools.

The sample size for WP 3 was determined using G Power statistical software. As there are no prior estimate of the outcome variables (serum level of ferritin, folate and VitB12, haemoglobin, weight, height and HDL) on grade one and two school children, the sample size was determined under the assumption of a statistical test (mean difference between two group), 95% confidence level, 80% power, conventional medium effect size of 0.5, a 1:1 ratio of intervention and control group and with ten total number of predictor variables. Accordingly, the estimated sample size became 128 (64 for each group). Then the sample size was adjusted considering a design effect of 2 since we are using a small cluster with a 10% attrition rate (Figure 3). The final total sample size was 282 (141 for the intervention and 284 for the control group).

### Sampling procedure for WP 2 and 3

**Figure 2.**
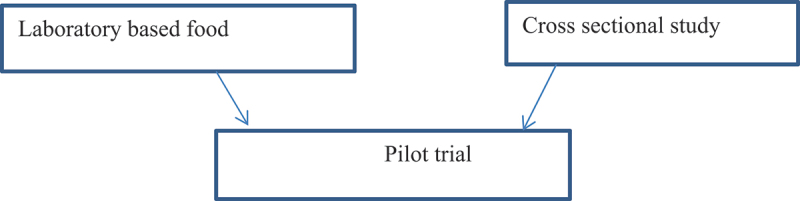
Study method relations.

A multi-stage stratified sampling technique will be used to select study participants. Six rural primary schools will be selected randomly for WP2, and individuals will be selected proportionally to the class size. After the cross-sectional study (WP 2), two primary schools with comparable characteristics will be selected from the six schools for WP 3. In this way, the pilot study will mimic a cluster randomized trial, where the intervention is the only difference between the participants. The school receiving the intervention or control intervention will be allocated randomly to the experimental and control groups. The food will be provided for the whole class, and the children who participated in the cross-sectional study and their caregivers will be invited to participate in the trial pilot (Figure 2).

## Work packages III and IV

The pilot experiment is described as a randomized trial with the following characteristics:

### PICO

**P** (Population): School children (7–10 years) of age in Sidama, Ethiopia

**I** (Intervention): Feeding of 200 g of nutrient and energy-dense amaranth plus flaxseed bread five days/week for four months

**C** (Comparison): Commonly used maize bread of a similar amount and colour to the bread given to the intervention group

**O** (Outcome): Nutritional anaemia (Ferritin, Iron, Haemoglobin, folate, vitamin B-12), HDL to test the effect of snack on healthy fatty acid, which is useful for growth. Growth (HAZ, BMI/age-change, WAZ) and education outcome (test score and dropout)

**T** (Time): 3 months

#### Eligibility

Inclusion Criteria: Accenting grade 1–2 actively, learning in school children (7–10 years old) with consenting parents or guardians at selected primary schools will be included.

#### Exclusion criteria

Those who are seriously ill, children with severe malnutrition, children with a history of any known allergy or participating in other trials, children who are unable to take solid food, grade 1 or 2 children aged > 10 years, and children with chronic illnesses like diabetes mellitus will be excluded from the pilot study.

#### Intervention and control food

The baseline data collection and meal preparation will take place at least two months before the anticipated start of recruiting trial participants, which is tentatively scheduled for 20 September 2024. The trial will then continue for four months, with the planned end date for follow-up being 20 January 2025.

The school will be assigned randomly by an external body by using lottery method to the trial groups: i. Amaranth plus flaxseed food, versus ii. 100% maize food.

The bread samples will undergo random inspections for weight and structure daily to ensure consistency. Additionally, at the midpoint of the intervention, a comprehensive analysis of the nutritional content of the bread will be conducted to assess any differences or changes resulting from the intervention. This approach will help maintain the integrity of the study and provide valuable insights into the nutritional impact of the bread produced in each group.

After allocation, the children will be given a card that contains their identification photo and attendance sheet. The trained feeding assistance personnel will deliver the food daily to the children at school during break time. They are also responsible for monitoring the children’s health for any unusual signs and measuring the amount of food left over (Table 2). The attendance sheet will have the place of signature for feeding assistance and the sign of leftover proportion to be filled out every day and at the end of the week, the checklist will be collected from the participant and evaluated by coordinators. The correct food will be provided five days per week for 4 months in each group.

**Table 2.** Schedule of enrolment, intervention and assessment of school children, Sidama, Ethiopia, 2024.

	May-July	August	September	October	November	December
**Preparation**						
Grain collection	X	X				
Flour preparation	X	X				
Data collectors training	X					
Pre testing	X					
Census (children registration)	X	X				
Informed consent	X		X			
**Enrolment Survey**						
Socio demographic data	X	X	X			
Haemoglobin level	X	X	X			X
Folate	X	X	X			X
Vitamin -B 12	X	X	X			X
CRP	X	X	X			X
Serum ferritin	X	X	X			X
AGP	X	X	X			X
Malaria	X	X	X			X
Stool examination	X	X	X			X
HDL	X	X	X			X
Anthropometry measure	X	X	X			X
Clinical examination	X	X	X	X	X	X
Illness history	X	X	X	X	X	X
Dietary assessment	X	X	X	X	X	X
**Intervention**						
Eligibility of child			X			
Randomization			X			
Deworming			X		X	
Feeding			Every day			
**Follow up visit**						
Clinical assessment			**Monthly**			
Illness history						
Food frequency data						
Compliance and adherence						X
Edline assessment						X

## Blinding

The trial will employ a double-blind design, meaning that the distributor will not know the contents of the food being distributed, and the data collectors will remain unaware of the trial allocations assigned to each school. Efforts were made to maintain this blinding by preparing the bread to have a similar appearance and texture. The coordinators will distribute the food according to the trial allocations, but they will not know which bread corresponds to the experimental or control groups, as each will be labelled with a school ID code (Page 11).

The food will be prepared based on the recommended dietary allowance (RDA), and according to RDA, the target daily intake of food will be 200 g for all children, which is considered to be comfortably consumed in one session. 200 g of the proposed food will be prepared according to the description in WP 1, with an assumed proportion close to 15% flaxseed + 20% amaranth + 40% wheat + 20% chickpea, also as an additive, 20 ml of sunflower oil and 10 g of sugar.

Both amaranth plus flaxseed food and 100% maize food will be prepared and packed separately. After packing, each package will be labelled with a pre-printed sticker containing the school’s name. In this way, we will ensure the distributor gets the right food for the right participant.

### Data collection in WP 2 and 3

Data collectors will receive training on the objectives of the study, data collection systems, interview techniques, micronutrient and anthropometric measurements, feeding procedures and field procedures. Prior to the data collection, standardization of micronutrient and anthropometric measures will be maintained.

### Pre-test

Pretesting of the questionnaire and food will be conducted in places away from where the actual study will be conducted. A pre-test will be conducted on 5% of the total sample size to check the tools. Materials for blood collection and anthropometry measures will be checked and prepared before starting the actual work.

### Interview

Using a pre-tested structured questionnaire, information on the socio-demographic (age, sex, educational status, level of income), disease history (diarrhoea, fever, vomiting, or breathing difficulties) within the last fifteen days and any history of chronic illness, repeated attacks of malaria and, recently diagnosed illness under medical treatment. Also, 24 hours dietary history of children will be gathered through face-to-face interviews with the children’s parents at baseline every month during the trial and at the end of the trial.

### Physical examination

Physical examination includes eye, mouth, neck, lymph nodes, skin, abdomen for enlargement of liver and spleen, extremities, pulse rate, respiratory rate, and temperature will be assessed by trained clinical health officers.

### Anthropometry

An electronic digital flat scale (Seca 874 weighting scale, made in Germany) will be used to measure body weight and height. Height will be taken using a Seca213 height board (Seca 213; Seca GmbH, Hamburg, Germany). Weight will be taken using a Seca 874 electronic flat scale (Seca 874; Seca GmbH, Hamburg, Germany) with the child barefoot, wearing light clothing after micturition. BMI-for-age will be used to measure obesity, overweight and thinness/wasting [44].

### Stool test

A Stool test will be done for work package two to identify factors associated with undernutrition.

Stool samples will be collected in clean, leak-proof stool cups using conventional methods. It will be tested shortly after collection using direct procedures. Direct microscopy to identify motile protozoa and the Kato-Katz stool test for the presence and severity of infection will be assessed for Ancyclostoma duodenale (hookworm), Ascaris lumbricoides (roundworm), and Trichuris trichiura (whipworm) ova.

### Blood collection and lab analysis

A 5–6 ml of venous blood will be collected via venepuncture, and then four millilitres will be transferred to a serum separator tube (SST) for biochemical analysis, and the remaining two will be drawn into to tube containing ethylene diamine tetraacetic acid (EDTA) for complete blood count (CBC) analysis. During blood collection, all infection prevention standards will be applied. The blood samples in the EDTA tubes will be wrapped in aluminium foil, continuously shielded from light during transportation to Hawassa Referral Hospital for CBC analysis.

The collected blood in SST will be allowed to clot for 30 minutes and centrifuged at 4000rpm for 10 minutes. After centrifuging, serum will be kept in a cryo vial tube with the participant’s identity and will be transported to Hawassa Referral Hospital using a cold chain carrier at −20°C. Biochemical analysis will be held at Hawassa Referral Hospital laboratory department using Cobas 6000 immediately (if possible) or stored at −70°C until analysis.

Haemoglobin will be determined using a complete blood count (CBC) machine (Sysmex Kx-21 automated analyser, Sysmex Corporation, Kobe, Japan) with a venous blood sample. Serum ferritin, iron, Vitamin B12, and folate will be analysed by electrochemiluminescence immunoassay (ECLIA) on Cobas 6000 (e601 module). The measuring principle for CRP and α 1-Acid glycoprotein (AGP) will be analysed using a particle-enhanced immunoturbidimetric assay and measured on Cobas 6000 (c501 module). Results will be determined via a calibration curve, which is an instrument specifically generated by 2-point calibration and a master curve provided via the reagent barcode.

To evaluate the impact of the Amaranth plus flaxseed food products on Education outcomes, measurements like enrollment, attendance, and test scores will be considered as outcome variables. School enrollment measures the student’s admission to a specific class and will be checked from the school’s list of students and their assignment to a specific class. The school attendance variable measures the rate of students’ absenteeism from school. The variable will be collected from the attendance sheet that will be registered every day before the day’s class starts. The test scores variable will be measured using the standard test exam results. Students will write Language, Mathematics, and General Sciences exam papers three times (Baseline, Mid-term, and end of intervention) during the intervention period.

The same exam papers will be used throughout the study. Progress will be monitored before, during, and after the intervention. Education professionals will prepare the exams, with a committee of four members per subject (12 total) trained to develop standard questions. A strict invigilation process will be enforced, with 10 trained invigilators from each school overseeing the simultaneous administration of exams for both control and treatment groups. Additional child and caregiver data will be sourced from the main project’s baseline and end-line surveys.

## Outcome measures variables

The study will report on: WP1: nutrient content; sensory attribute of formulated food; WP2: prevalence and causes of nutritional anaemia (serum iron, haemoglobin, serum ferritin, vitB12, folic acid); level of high-density lipoprotein (HDL) to look at the effect of healthy fat intake from flaxseed mix; and WP3: change in nutritional anaemia (serum iron, ferritin, folate and Vit B-12), change in HAZ, BMI/age, WAZ and HDL, and acceptability of amaranth plus flaxseed food are the outcome variables of this study.

## Statistical data analysis:

The statistical software SPSS version 27 and STATA version 16 will be used for data analysis. To determine if the data are normally distributed, a one-sample Kolmogorov – Smirnov test will be used. Mean and standard deviation will be reported for normally distributed data, while median and interquartile ranges will be reported for non-normally distributed data. Parametric analysis will be used to analyse data that will be normally distributed, and nonparametric analysis will be used to analyse data that will not be normally distributed. One-way ANOVA will be used to determine the significance of the mean difference of scores for colour, appearance, flavour, taste, consistency, mouth feel, and overall acceptability (work package I), and also to compare the serum levels of trace elements among the participants (work package II and III). The mean score of proximate composition, mineral, and phytate will be analysed by using the mean difference to determine the significant change. Each determination of nutrient measurement will be carried out on three separate samples and will be analysed in triplicate (work package I). Using the WHO Anthro-Plus version 10.4 software, the z-score values for height, weight, and BMI for age will be determined (work packages II and III). To assess the effects of energy and nutrient-dense amaranth-based food on nutritional status and educational attainments, intention-to-treat (ITT) analysis will be used (work package III). Data will be presented as frequency, percentages, mean, standard deviation, and mean difference. A p-value of < 0.05 will be statistically significant in all analysis (work packages I-III).

**Figure 3.**
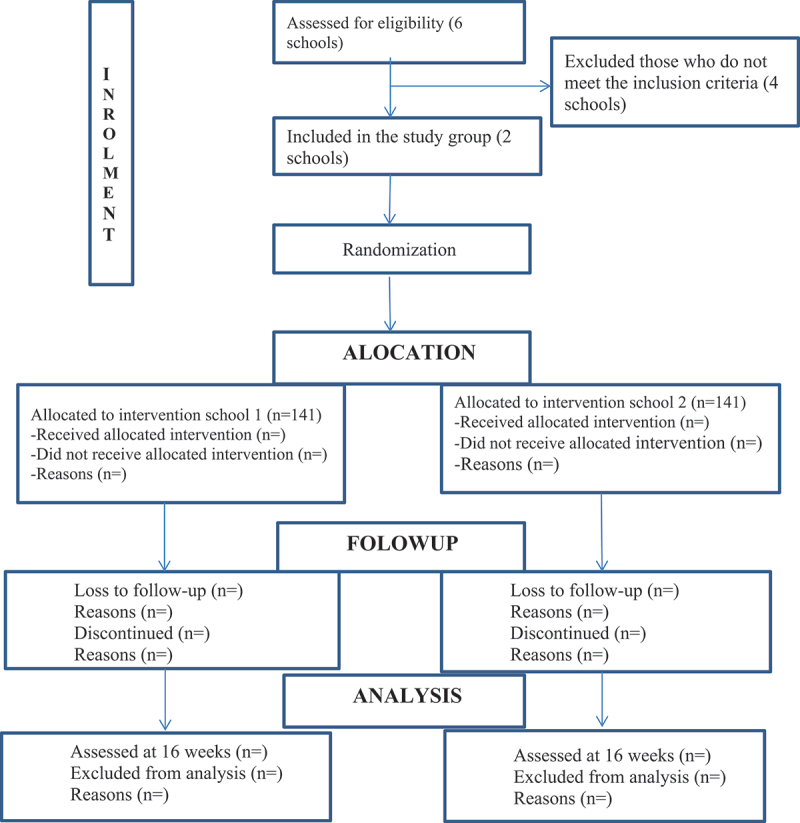
Trial profile. Abbreviation BMI: Body Mass Index, EDHS: Ethiopian Demographic Health Survey, HAZ: Height for Age Z-score, HDL: High-Density Lipoprotein, HGSF: Home-Grown School Feeding, FRESH: Focusing Resources on Effective School Health, IDA: Iron Deficiency Anaemia, ICC: Intra-Class Correlation Coefficients, MUAC: Mid-Upper Arm Circumference, RDI: Recommended Dietary Intake, SHN: School Health Nutrition, USDA: United States Department of Agriculture, WAZ: Weight for Age Z-score, WFP: World Food Program.

## Discussion

School feeding programs are vital for mitigating undernutrition among school children. Despite their importance, these programs in rural areas of the country often fail to effectively reduce malnutrition rates among school-aged children [31]. However, international systematic reviews indicate that school feeding interventions can lead to positive weight changes compared to non-feeding groups.

The proposed amaranth and flaxseed snack offers a nutrient-dense, easy-to-prepare, and distribute alternative that could significantly differ from commonly implemented food options in terms of cost and accessibility. The findings from this study may inform policymakers about the value of amaranth, which is currently not recognized as a significant crop at the Ministry of Agriculture level, and flaxseed, which has not been used to complement school snacks. This could encourage policymakers involved in school feeding programs to consider incorporating these ingredients to enhance the nutritional content of locally available food groups, thereby improving children’s nutritional status while also addressing cost and sustainability for potential scale-up.

While this study may face challenges related to financial constraints, we are actively seeking adequate funding to ensure its completion. Additionally, food acceptability may pose another challenge; however, previous studies indicate that combining amaranth with other ingredients has shown good acceptability among children. To assess further, we will conduct a pre-test on the acceptability of the proposed snack.

The generalizability of this study extends to similar settings in Ethiopia and other regions facing comparable nutritional challenges, particularly where similar agricultural products are available. The snack will be developed using locally sourced grains, enhancing its relevance and applicability.

Should the outcomes of this study prove ineffective, we recommend conducting further research to explore alternative approaches. The use of amaranth and flaxseed in school feeding programs is sustainable due to their local availability; amaranth is drought-resistant and yields higher than conventional staple cereals. This may encourage farmers to cultivate amaranth, promoting its use at the household level and potentially improving food security. While the cost of the proposed snack may be lower than that of conventional programs, further studies are necessary to assess its cost-effectiveness comprehensively.

## Supplementary Material

Consent form.docx

## Advantages and disadvantages of the research for the community and society

This research holds no foreseeable disadvantages for the community or society. It will benefit the community by highlighting the potential of amaranth and flaxseed as a nutritious food source, potentially increasing farmer income and improving household food security. The findings may also influence policymakers to consider amaranth and flaxseed as alternative food options for school feeding programs, particularly in drought-prone areas. In the long run, it could enhance local agricultural practices and food security by promoting the cultivation of amaranth, a drought-resistant and more productive grain. In the long run, it could improve local agricultural practices and food security by practising amaranth cultivation, which is drought-resistant and a more productive grain.

The research will contribute to scientific knowledge regarding the benefits of amaranth and flaxseed for preventing undernutrition. Overall, the potential benefits of this research outweigh the minimal risks involved. The findings will be published openly to support policy decisions and further scientific inquiry.

## Amendment

Regarding protocol modifications, communication will be made to relevant stakeholders, including the IRB/IEC and sponsor.

## Auditing

The study welcomes any interested organization, whether it be governmental or non-governmental, to conduct an audit.

## Confidentiality

Participant information will be stored in a secure location and only accessible to authorized personnel, and participant identities will be kept anonymous. Data will be anonymized when transferred to analysis and for data sharing with third parties.

## Dissemination

The result of this study will be disseminated through Internal Seminars, Conference presentations using outreach and public engagement events. Regular reporting and communication to stakeholders will be done. It will also be disseminated through Publications.

## Individual advantages and disadvantages from the research

This research aims to benefit all participants. The amaranth/flaxseed group will receive a nutrient-rich diet, while the maize group will benefit from increased calorie intake. The study offers a chance to identify and potentially treat underweight and anaemia in children. Participants with severe conditions will be referred to a hospital for further care.

All children will receive anaemia treatment based on their needs at the study’s conclusion. Regular monitoring will identify and address any severe nutritional problems, with hospital referrals if needed. Monthly health education sessions will promote healthy habits and feeding practices. While some discomfort might occur during sample collection, standard precautions will be taken to ensure safety.

Minimal discomfort may be experienced from the venipuncture; however, we will implement standard precautions to ensure safety during the procedure. We will closely monitor for any potential food allergies and adverse reactions, conducting daily follow-ups to identify any negative effects on the children.

## Trial status

The trial was registered with the Pan-African Clinical Trial Registry on 28 March 2024, and the trial number is PACTR202403614331028. This paper has a preprint version at https://medrxiv.org/cgi/content/short/2025.2 May 25321679v1

## Appendix

The purpose of the study is to reduce under-nutrition among school children by promoting amaranth plus flaxseed products from locally grown, standardised foods in Ethiopia.

This study is a part of a nutrtion PhD project of Hawassa University that works on to produce nutrient rich food for school going children.

Your child is selected randomly to be involved in this study without criteria. If you are willing to participate, you will be expected to realize the following requirement you are required to give us an information. The study has two phase of data collection it is before starting the intervention and at the end of the intervention. During this two data collection phase you expected to give an information on the sociodemographic, dietery history, houshold food insecurity and form your child weight and height, blood sample and stool sample will be collected to know the child nutrtional status. Then the child will feed amaranth plus flax seed food or maize only food every day for four month. The allocation of the intervention is random.

**The potential disadvantage of the study will be** the child will fell mild pain during blood collection and discomfort stool sample collection there is no further physical or psychological risk expected being involved in the study.

**The advantgae** of the study is you have the right to know the finding of the study, you will be given experts advice about nutrtional problem and nutrient rich food. The result which produced from your participation will help to fight nutrtional problems in school going childrens.

**Participation** in the study is voluntary. You can withdraw your consent to participate in the study at any time and without stating any particular reason. This will not have any consequences for your further child treatment.

If you agree to participate in the study, you are entitled to have access to the information registered about you. You are further entitled to correct any mistakes in the information we have registered. If you withdraw from the study, no further information or material will be collected about you. Data that have already been collected will not be deleted

**Confidentiality**: the samples and data that are registered about you will only be used in accordance with the purpose of the study as described above. All the data and samples will be processed without name, personal identification number or other directly recognisable type of information. A code number links you to your data and samples through a list of names. The list that can link your name to the code number will be stored at Hawassa University data registry port only, and only the authorised study staff will have access to this list.

The data will be destroyed 15 years after the final report is published.

It will not be possible to identify you in the results of the study when these are published.

If you wish to participate, sign the declaration of consent on the final page.

If you have questions concerning the study, you may contact study supervisor Alemselam Zebdewos Orsango with cell phone 0911000961 or e-mail address zalemselam@yahoo.com

Please tell us if you agree or not

Yes ______________

No______________

Mothers name _________________Signature _____________

Fathers name _________________Signature _____________

Child name ___________________Signature _____________

Data collector name and signature ______________________________

Date _____________________________

Thank you for your willingness to participate in this study
