# Supplementary material for: Improving school-age nutrition and school performance through amaranth plus flaxseed food product distribution in Sidama, Ethiopia: a study protocol
Source: Glob Health Action. 2025 Oct 15;18(1):2556087. doi: 10.1080/16549716.2025.2556087 (PMC12529742; doi:10.1080/16549716.2025.2556087)
Supplement: Consent form.docx [file ZGHA_A_2556087_SM1846.docx]

**Appendix-I**


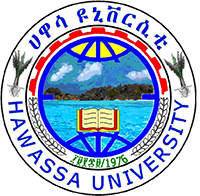


**Improving school agenutrition and school performance through amaranth plus flaxseed food product distribution in Sidama, Ethiopia**

The purpose of the study is to reduce under-nutrition among school children by promoting amaranth plus flaxseed products from locally grown, standardised foods in Ethiopia.

This study is a part of a nutrtion PhD project of Hawassa University that works on to produce nutrient rich food for school going children.

Your child is selected randomly to be involved in this study without criteria. If you are willing to participate, you will be expected to realize the following requirement you are required to give us an information. The study has two phase of data collection it is before starting the intervention and at the end of the intervention. During this two data collection phase you expected to give an information on the sociodemographic, dietery history, houshold food insecurity and form your child weight and height, blood sample and stool sample will be collected to know the child nutrtional status. Then the child will feed amaranth plus flax seed food or maize only food every day for four month. The allocation of the intervention is random.

**The potential disadvantage of the study will be** the child will fell mild pain during blood collection and discomfort stool sample collection there is no further physical or psychological risk expected being involved in the study.

**The advantgae** of the study is you have the right to know the finding of the study, you will be given experts advice about nutrtional problem and nutrient rich food. The result which produced from your participation will help to fight nutrtional problems in school going childrens.

**Participation** in the study is voluntary. You can withdraw your consent to participate in the study at any time and without stating any particular reason. This will not have any consequences for your further child treatment.

If you agree to participate in the study, you are entitled to have access to the information registered about you. You are further entitled to correct any mistakes in the information we have registered. If you withdraw from the study, no further information or material will be collected about you. Data that have already been collected will not be deleted

**Confidentiality:** the samples and data that are registered about you will only be used in accordance with the purpose of the study as described above. All the data and samples will be processed without name, personal identification number or other directly recognisable type of information. A code number links you to your data and samples through a list of names. The list that can link your name to the code number will be stored at Hawassa University data registry port only, and only the authorised study staff will have access to this list.

The data will be destroyed 15 years after the final report is published.

It will not be possible to identify you in the results of the study when these are published.

If you wish to participate, sign the declaration of consent on the final page.

If you have questions concerning the study, you may contact study supervisor Alemselam Zebdewos Orsango with cell phone 0911000961 or e-mail address [zalemselam@yahoo.com](mailto:zalemselam@yahoo.com)

Please tell us if you agree or not

Yes ______________

No______________

Mothers name _________________Signature _____________

Fathers name _________________Signature _____________

Child name ___________________Signature _____________

Data collector name and signature ______________________________

Date _____________________________

Thank you for your willingness to participate in this study
